# Medullary carcinoma of the breast: Role of contrast-enhanced MRI in the diagnosis of multiple breast lesions

**DOI:** 10.2349/biij.5.4.e27

**Published:** 2009-10-01

**Authors:** SN Abdul Rashid, K Rahmat, KJ Jayaprasagam, K Alli, F Moosa

**Affiliations:** 1 Department of Radiology, Universiti Putra Malaysia, Selangor, Malaysia.; 2 Department of Biomedical Imaging, University of Malaya, Kuala Lumpur, Malaysia

**Keywords:** Fibroadenoma, medullary carcinoma breast, ultrasonography

## Abstract

Medullary carcinoma is a rare breast carcinoma with a syncytial growth pattern and high-grade cytology. It can be difficult to diagnose and may be missed on conventional imaging as the findings may overlap with benign lesions i.e. fibroadenomas. The authors report a case of a 25-year-old female who presented with multifocal breast lumps diagnosed with medullary carcinoma and fibroadenomas. Imaging and pathological correlation with contrast-enhanced MRI are presented in the diagnosis of these lesions.

## INTRODUCTION

Medullary carcinoma of the breast is a rare subtype of invasive breast carcinoma with a high-grade cytology but has been reported to have a good prognosis. It is an uncommon type of invasive breast cancer accounting for 5 - 7% of all breast cancers, occurring most frequently in women in their late 40s and early 50s. It is also more common in women who have a BRCA1 mutation. Radiologically, it can be difficult to diagnose and may be missed as the imaging findings may overlap with benign lesions i.e. fibroadenomas on conventional mammography and ultrasonography. The authors report a case of a 25-year-old female who presented with multifocal breast lumps diagnosed with medullary carcinoma and fibroadenomas. Imaging and pathological correlation with contrast-enhanced MRI are presented in the diagnosis of these lesions. This case highlights the usefulness of utilizing contrast-enhanced MRI in the characterization of multiple breast lesions when conventional imaging findings are equivocal.

## CASE REPORT

A 25-year-old female presented to the Breast Physician in April 2007 with a 2-month history of a painless lump in the right breast. She had no associated nipple discharge or other constitutional symptoms. Physical examination revealed a 3 cm, non-mobile mass in the upper outer quadrant of the right breast. It was non-tender and the overlying skin was normal. There was no axillary lymphadenopathy. Prior to this presentation, the patient first presented to a private medical center with bilateral breast lumps in 2002. A few of these lumps were surgically removed and histologically proven to be fibroadenomas. During this period, her maternal aunt had developed breast cancer and was undergoing treatment.

Breast examination and ultrasound follow-up was performed every 12 months. She was symptom free until she developed a painful lump in the left breast in 2004. As this lesion clearly demonstrated typical benign features characteristic of a fibroadenoma, the lesion was followed up with ultrasound and had remained essentially unchanged for the next 3 years. She subsequently developed a new lump in the right breast for which she presented to us.

**Figure 1:**
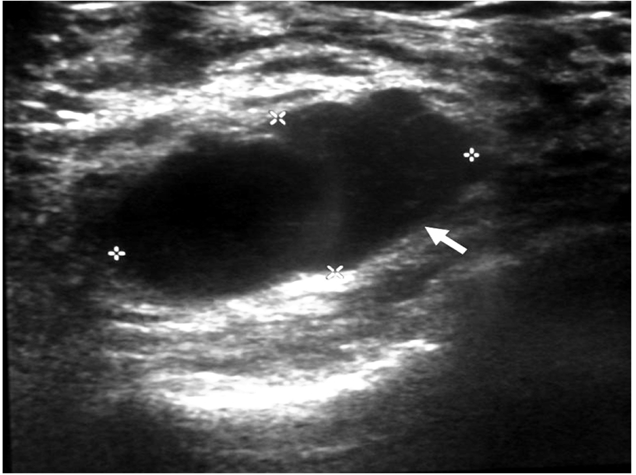
Ultrasound examination of the right breast showing a well circumscribed hypoechoic mass (white arrow) with internal echogenicity and acoustic enhancement with lobulated margins.

**Figure 2:**
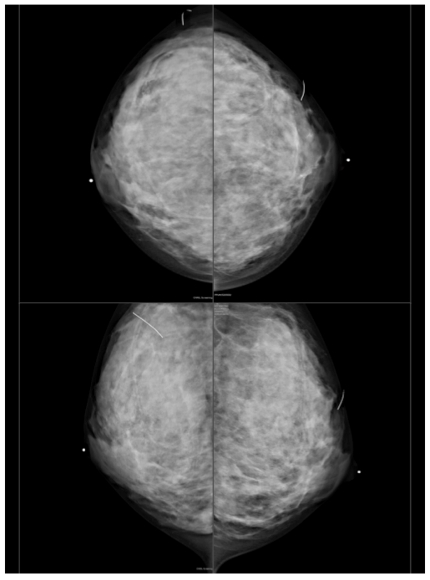
Mammograms showed dense breast parenchymal pattern limiting the sensitivity of the examination. No suspicious masses, architectural distortion or microcalcifications were present.

Ultrasound examination of the right breast demonstrated a well-circumscribed lobulated hypoechoic mass with smooth margins corresponding to the palpable lump at the 9 o’clock position (Figure 1). There were inhomogeneous internal echoes associated with posterior enhancement which was suggestive of proteinaceous materials or internal haemorrhage. Colour Doppler imaging showed some demonstrable internal vascularity. On the basis of these morphological features, the lesion was categorized as suspicious finding BI-RADS 4. The previously known stable nodule in the left breast had remained unchanged in size and appearance; however two new benign looking lesions had been identified. Bilateral mammograms was subsequently performed which was of limited diagnostic value due to the dense breast parenchymal pattern (Figure 2). No microcalcifications or axillary lymphadenopathy was demonstrated. The fine needle aspiration biopsy (FNAC) performed under ultrasound guidance was consistent with medullary carcinoma.

**Figure 3:**
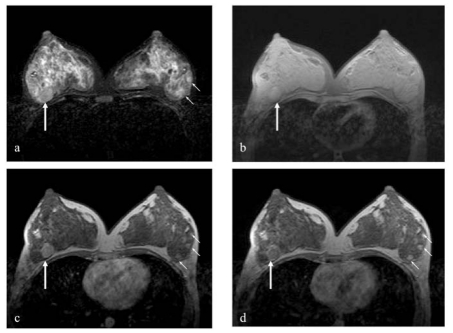
MRI study of the breasts showing the medullary carcinoma in the right breast adjacent to the pectoralis muscle (long white arrow) appearing (a) isointense on T2W STIR axial (TR: 5450, TE: 60), (b) isointense on T1W fat sat axial sequence (TR: 11.2, TE: 5), (c) demonstrated homogenous enhancement during the early dynamic post-gadolinium sequence at 1 minute, and (d) delayed peripheral enhancement during the dynamic post-gadolinium at 6 minutes, suggestive of a malignant lesion. Note the smaller three enhancing fibroadenomas in the left breast (short white arrows) in (c) and (d), which demonstrated gradual homogenous enhancement throughout the dynamic phases.

**Figure 4:**
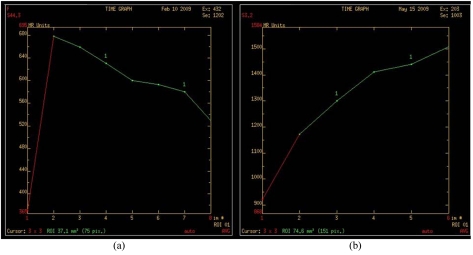
Dynamic contrast-enhanced time intensity curves of (a) the right breast mass with early wash-in and early wash-out pattern, (b) the benign left breast lesions with gradual enhancement pattern throughout the dynamic phases.

Contrast-enhanced MRI (Siemens 1.5 Tesla), which was performed to exclude multifocal / multicentric carcinomas, showed a corresponding lobulated mass with smooth margins in the right upper outer quadrant. (Figure 3). The lesion was deep seated, lying adjacent to the pectoralis major with no evidence of infiltration into the underlying muscles. It demonstrated rapid homogenous enhancement in the early dynamic phase with an early washout pattern (Figure 4a). The other three nodules seen in the left outer quadrant showed gradual persistent enhancement throughout the dynamic study which were in favour of benign pathology (Figure 4b). The examination concluded the presence of a solitary right breast carcinoma with multiple left breast benign lesions. At the request of the surgeon, core biopsies were also performed on all the three lesions in the left breast under ultrasound guidance which were confirmed to be fibroadenomas.

**Figure 5:**
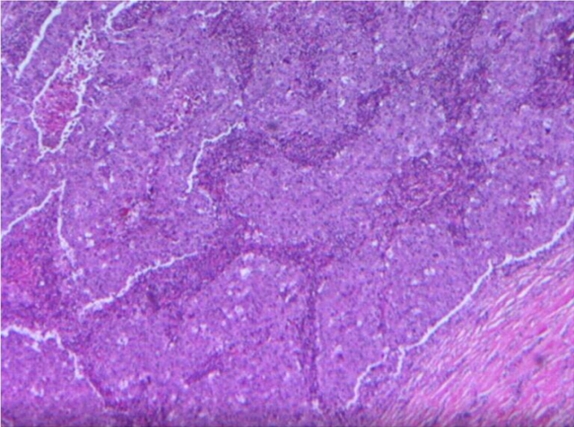
Histopathology slide showing lymphocytic infiltration with solid sheets of large anaplastic cells with syncytial growth.

After discussing the treatment options with the patient, breast conservation surgery of the right breast with axillary clearance was performed in June 2007. Gross examination of the resected specimen revealed a smooth, well circumscribed solid mass measuring 2.0 cm × 1.8 cm with bloody foci. It also had a fairly homogenous appearance with lack of dense fibrous stroma. Histopathological examination revealed lymphocytic infiltration with solid sheets of large anaplastic cells and syncytial growth consistent with medullary carcinoma (Figure 5).

## DISCUSSION

Medullary breast carcinoma is a rare breast malignancy comprising less than 5% of breast carcinomas in majority of studies [1]. It has a predilection for women of a younger age group and in a study by Rosen *et al.* [2] it was found to constitute 11% of all breast malignancies among women aged 35 and younger. Some studies have revealed a similar degree of prognosis between medullary carcinoma and infiltrating ductal carcinoma of the same stage [3-5] whereas others have observed equal survival rates among these two types of carcinoma [6,7].

Medullary carcinoma is a subtype of infiltrating ductal carcinoma. Therefore, the radiographic features are indistinguishable from the other tumours in the same subgroup. On gross examination, these groups of tumours are often well circumscribed with a median size of about 2.0-3.0 cm. It is a moderately firm, discrete tumour with a lobulated or nodular cut surface and may have necrosis or haemorrhagic changes [8]. Histologically, medullary carcinoma demonstrates syncytial growth pattern of poorly differentiated tumor cells with a high mitotic rate. Prominent lymphocytic infiltrates with a circumscribed microscopic appearance of desmoplastic inflammatory reaction involving mainly the periphery was also diffusely present throughout the substance of the tumor - this is another characteristic feature, which may account for its clinical and biological behaviour [9].

Normally, no glandular or fatty breast tissue should be found within the invasive portion of the tumour. IgG cells predominate with many T-lymphocytes and more than 90% of these tumours are estrogen- and progesterone-receptor negative [9]. Histopathological examination in this patient revealed lymphocytic infiltration with solid sheets of large anaplastic cells and syncytial growth consistent with medullary carcinoma. Medullary carcinoma of the breast is considered to carry a more favourable or equal prognosis compared to other subtypes of infiltrating ductal carcinoma [9]. This is a biological paradox because its clinical behavior contrasts with its anaplastic morphology. The main characteristic feature of a medullary carcinoma is the dense lymphocytic infiltrate. The presence of increased numbers of activated cytotoxic lymphocytes in medullary carcinoma of the breast may be a key mechanism active in the host versus tumour response leading to improved prognosis.

Based on several studies done previously, medullary carcinoma of the breast is usually described as an irregular shaped mass with microlobulation and least frequently with posterior acoustic shadowing sonographically [10]. Calcification is usually not present on mammographic imaging. Sonographic features of medullary carcinoma and other subtypes group do not differ substantially.

In a study conducted by Cho and colleagues, they found that their cases of medullary carcinoma were larger with a greater L/AP ratios than the fibroadenomas [11]. Their medullary cancers were round or lobular in shape and demonstrated thick walls with anechoic cystic space. Medullary carcinomas generally demonstrate a lesser degree of sound attenuation as compared to other malignant tumours on ultrasound. Other sonographic features include smooth contours, weak internal echoes and oval or round shape [12]. Typical fibroadenomas appear well circumscribed, homogeneous, hypoechoic with edge shadowing and gentle lobulations with occasionally coarse calcification within [13]. It is also a recognized fact that medullary carcinoma mimics a fibroadenoma sonographically [13].

In this patient, the medullary carcinoma was shown on ultrasound to be a well-defined lobulated hypoechoic mass with posterior acoustic enhancement, or increased through transmission deep to it. This finding may be due to a homogenous population of tumor cells in the mass that have few acoustic interfaces, which would facilitate the transmission rather than the reflection of ultrasound. On mammogram, medullary carcinoma commonly appears uniformly dense, round or oval, non-calcified and with lobulated margins [12]. The width of the lobulations measured at the base, ranged from 2 to 3 mm, in some cases larger, solitary or multiple indentations were noted [12]. Some lesions, exhibited a halo sign at mammography, which until recently was considered strong evidence of a benign lesion [12]. In this patient, the lesion was not visualised on mammogram due to the dense stromal pattern that had obviously obscured the cancer as well as the fibroadenomas. No axillary lymphadenopathy was seen in this patient either.

Young *et al.* [8] have reported cases of bilateral medullary carcinomas in about 3-18% of patients as well as multicentric medullary carcinoma in one breast [8, 10]. In this case, MRI of both breasts was performed to rule out multiplicity as she had a total of 4 lesions in both breasts. However, only one malignant lesion was identified in the right breast. The washout enhancement pattern on the dynamic study combined with the lesion margin is regarded as a highly specific diagnostic tool in characterizing the lesion as malignant. Peripheral rim enhancement demonstrated in this lesion was also strongly associated with malignancy.

The other lesions were shown to have benign MR features based on the morphology and pattern of enhancement. It is difficult to differentiate the different subtype of infiltrating ductal carcinoma based on MRI findings alone. Majority of the lesions showed a strong or moderate degree of enhancement with peripheral enhancement and an early washout enhancement pattern in the dynamic sequence [14]. However, there are no specific features to suggest medullary carcinoma based on these findings, therefore other supportive features like size of lesion, margin, lobulation and correlation with ultrasound and mammogram are useful supporting parameters [15, 16]. At the time of writing this article, the protocol of breast MR that had been used in the authors’ institution was as follows:

1. Axial T1W fat sat
2. Axial T2W PD/STIR
3. Sagittal T2W STIR
4. Dynamic post-gadolinium T1W (Axial) study with 1 minute intervals
5. Axial post-gadolinium T1W fat sat

Fibroadenomas are benign breast tumours that represent proliferative process in the ducts and are considered to be an aberration of the normal breast development. They are not considered to have any malignant potential. However, in a study done by Dupont *et al*, they found that the risk of invasive breast cancer was twice as likely to occur in patients with fibroadenomas [17]. The relative risk was also found to be higher in women who have atypical fibroadenomas, proliferative disease, or a familial history of breast cancer. Although there is no literature pertaining to the association of medullary carcinoma of the breast and fibroadenomas, this case illustrates an example of how a medullary carcinoma can mimic a fibroadenoma. Follow up and good clinical history correlation is recommended for any patients on follow up for fibroadenomas. Any fibroadenomas with significant increase in size or develops atypical features on follow up or measures more than 3-4 cm in diameter should be surgically excised and biopsied regardless of patient’s age and results of triple testing. [13]. In this case, the patient had a family history of breast carcinoma and multiple fibroadenomas, both of which were risk factors in the development of breast carcinoma.

The 5-year survival rate for medullary carcinoma is approximately 78% [9]. Death secondary to this disease is only 10% though. The 20-year disease free survival for stage I and II patients is approximately 95% and 61%, respecti vely [9]. Breast conservation surgery is the treatment of choice for lesions less than 3.0 cm with no more than 3 nodes involved. A right breast conservation surgery and axillary clearance was done for this patient. She is currently on chemotherapy and is to be followed by course of radiotherapy. In conclusion, this case demonstrates the importance of MR correlation to characterize breast lesions and exclude multiplicity of malignant lesions before surgery.
